# Comparative evaluation of artificial saliva and complete artificial saliva as solvent vehicles for *in vitro* toxicity testing of oral tobacco products

**DOI:** 10.3389/ftox.2025.1657073

**Published:** 2025-12-08

**Authors:** Xuefei Cao, Mariana T. Farcas, Yevgeniya V. Prepelitskaya, Abaigeal Ritzenthaler, Jennifer Molignano, Jonathan Oldach, Marisol M. Gutierrez

**Affiliations:** 1 Altria Client Services LLC, Richmond, VA, United States; 2 MatTek Corporation, Ashland, MA, United States

**Keywords:** artificial saliva, complete artificial saliva, smokeless tobacco, extraction efficiency, mechanistic

## Abstract

**Background:**

Oral tobacco-derived nicotine (OTDN) pouches are tobacco-free products and are considered potential reduced-risk alternatives to traditional tobacco products within the tobacco harm reduction framework. Despite their growing popularity, the local oral toxicity profiles of OTDNs remain poorly characterized. Although *in vitro* toxicity studies have been conducted, the lack of standardized testing protocols, including solvent vehicle selection, limits data comparability across laboratories. To address this, we evaluated and compared the biological effects of artificial saliva (AS) and complete artificial saliva (CAS) across four *in vitro* oral models to identify an appropriate solvent vehicle for mechanistic testing.

**Methods:**

Extraction efficiency was first assessed using the Swedish-style reference snus pouch CRP1.1 extracted in AS and CAS from 10% to 30% w/v concentrations. Nicotine and tobacco-specific nitrosamines (TSNAs) were quantified for extraction efficiency. The biological effects of AS and CAS, including cytotoxicity, oxidative stress, and inflammatory responses, were evaluated across four *in vitro* oral models, including monolayer normal human gingival fibroblasts (NHGFs) and oral epithelial cells (NHOEs), as well as 3D organotypic models (EpiOral™ and EpiOral™ Full Thickness).

**Results:**

The results showed comparable nicotine extraction efficiency between AS and CAS, with no significant impact from pouch cutting size or extraction duration. However, AS demonstrated higher efficiency in extracting TSNAs compared to CAS at 10% and 20% w/v, with the extraction efficiency decreasing as extract concentrations increased.

Neither AS nor CAS induced cytotoxicity in any of the oral models. CAS triggered oxidative stress at the highest concentration in ORL-300-FT. Both AS and CAS elicited concentration-dependent pro-inflammatory responses in NHGFs and NHOEs. Specifically, AS increased both IL-6 and IL-8 secretion in NHOEs, while CAS elevated IL-8 release in both NHGFs and NHOEs but exhibited opposing effects on IL-6 secretion in NHOEs. In the organotypic tissue models, both AS and CAS reduced IL-6 secretion without significantly affecting IL-8 levels.

**Conclusion:**

These findings emphasize the importance of evaluating additional biological responses alongside cytotoxicity in vehicle control studies. CAS and ORL-300-FT were chosen for future testing due to their minimal vehicle effects and greater biological relevance, providing a robust platform for assessing oral tobacco product toxicity.

## 1 Introduction

Oral tobacco-derived nicotine (OTDN) products have rapidly gained popularity among tobacco product consumers seeking smoke-free and tobacco-free alternatives for nicotine delivery. These products typically consist of nicotine, food-grade flavorings, plant-based fibers or fillers, and other additives, and are designed to be placed between the gum and lip, allowing nicotine absorption through the oral mucosa. Their growing appeal as potentially reduced-risk products is largely attributed to the absence or substantially reduced levels of the harmful and potentially harmful constituents (HPHCs) commonly found in smoke from combustible cigarettes and traditional smokeless tobacco (ST) products (Azzopardi et al., 2022; Jablonski et al., 2022; Wagner et al., 2022).

Despite their increasing adoption, the toxicological profile of OTDN products, particularly their local (oral), long-term health effects, remains insufficiently studied. The current status underscores the need for robust toxicity evaluation strategies to assess their safety and potential risks. *In vitro* toxicology studies have emerged as indispensable tools for evaluating product safety, including that of oral tobacco products (OTPs), by providing valuable insights into cellular responses such as cytotoxicity, oxidative stress, and inflammatory responses (East et al., 2021; Keyser, 2022; Rinaldi et al., 2023; Shaikh et al., 2022; Yu et al., 2024; Yu et al., 2022). However, unlike combustible cigarettes, the current state of *in vitro* toxicity evaluation for OTDNs faces a multitude of challenges, including the selection of physiologically relevant solvent vehicles and *in vitro* test systems that better represent the oral environment, as well as the lack of standardized testing protocols. To address these gaps, advanced *in vitro* methodologies that better replicate the environment in the oral cavity are essential for a more comprehensive understanding of the potential disease risks associated with OTDN usage. As regulatory agencies and public health organizations strive to evaluate the safety of these products, robust *in vitro* toxicology studies will play a pivotal role in informing risk assessments and guiding policy decisions.

Although an increasing number of *in vitro* studies on OTDN products have been published, there is currently no consensus or standardized methodology for preparing test materials from these products. Differences in extract concentrations, solvent vehicles, extraction temperatures and durations, as well as centrifugation speeds, have been reported (Bishop et al., 2020). The lack of standardized methods presents a significant challenge to ensuring data comparability across laboratories. To address this issue, members of the Cooperation Centre for Scientific Research Relative to Tobacco (CORESTA) *In Vitro* Working Group are actively evaluating the similarities and differences among existing sample preparation methods, with the goal of developing comprehensive guidelines for OTDN test material preparation in *in vitro* testing (Moses, 2024).

Among the variables under evaluation, the selection of an appropriate solvent vehicle for OTPs is particularly critical for enhancing the biological relevance of *in vitro* toxicological findings. Various solvent vehicles have been reported for extracting OTDN and ST products for *in vitro* toxicity testing, including phosphate-buffered saline (PBS), cell culture media, dimethyl sulfoxide (DMSO), artificial saliva (AS), and complete artificial saliva (CAS) (Arimilli et al., 2012; Bishop et al., 2020; Coggins et al., 2012; Gao et al., 2013; Misra et al., 2014; Rickert et al., 2007; German Institute for Standardization, 2002; Zanetti et al., 2019). Among these solvent vehicles, AS (German Institute for Standardization, 2002) and CAS (Chou and Hee, 1994; Keyser, 2022) contain inorganic compositions that are similar to those found in human saliva. However, CAS also consists of essential salivary components, such as enzymes, mucins, glucose, and urea, making it a closer mimic of human saliva. Therefore, CAS is considered to provide greater biological relevance than AS and other solvent vehicles.

**FIGURE 1:**
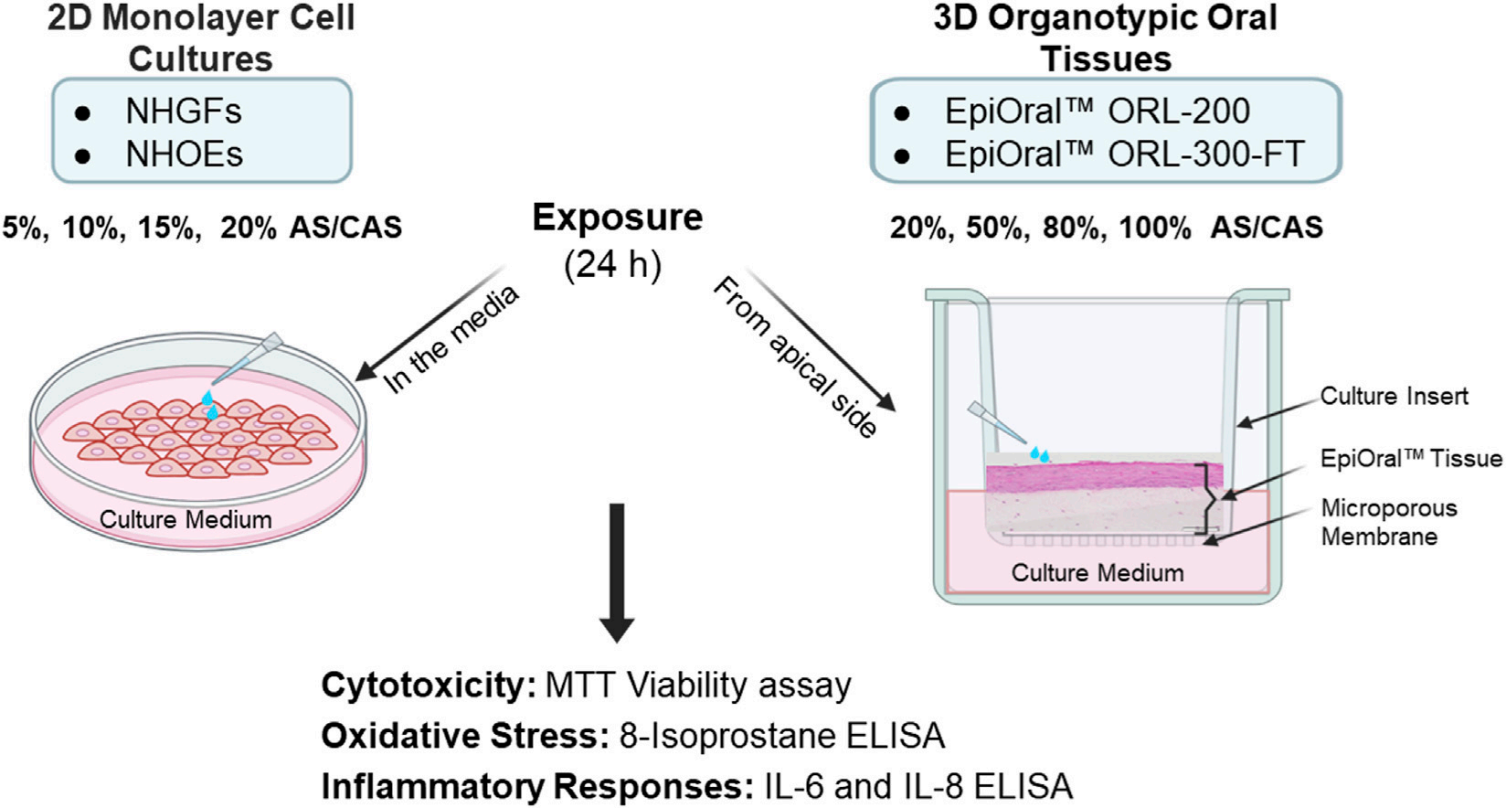
Schematic representation of the experimental design for 2D monolayer primary cells and 3D organotypic oral mucosal models. Different dilutions of AS and CAS were applied either to the culture vessels of the 2D cells or to the apical surface of the 3D oral mucosal models. Following a 24-h exposure, cytotoxicity, oxidative stress, and inflammatory responses were evaluated.

In this study, we aimed to identify an *in vitro* testing platform with greater biological relevance for future oral tobacco product assessments. To achieve this goal, we compared the effects of AS and CAS as solvent vehicles across four *in vitro* oral models representing varying levels of biological complexity (see Materials and Methods for details). To minimize donor-to-donor variability, all four *in vitro* oral models were derived from the same donor. 3D oral mucosal models have been widely employed to evaluate the oral toxicity of combustible cigarettes and alternative modified-risk tobacco products, including heated tobacco products, electronic cigarettes, and oral tobacco products (Keyser, 2022; Klausner et al., 2021; Zanetti et al., 2019) to demonstrate the reduced risk potential of these products compared to combustible cigarettes. As a crucial and integral step in the *in vitro* testing process, extraction methods were first optimized using the CORESTA snus reference product CRP1.1 to determine the achievable and acceptable maximum extract concentrations for OTPs. Following the optimization of extraction methods, the effects of varying concentrations of AS and CAS on cytotoxicity, oxidative stress, and inflammatory responses, molecular factors known to contribute to oral disease development (Keyser, 2022; Zhang Y. et al., 2019), were measured and compared across all four *in vitro* oral models (Figure 1).

## 2 Materials and methods

### 2.1 Reagents

Triton™ X-100, Dulbecco’s Phosphate-Buffered Saline (DPBS), transepithelial electric resistance (TEER) Buffer-GLC, and the MTT kit were provided by MatTek Corporation (Ashland, MA). Phorbol 12-myristate 13-acetate (PMA), ionomycin, Human IL-8/CXCL8 DuoSet ELISA, and Human IL-6 DuoSet ELISA were purchased from R&D Systems (Minneapolis, MN). The LDH Cytotoxicity Detection Kit, Butylated Hydroxytoluene (BHT), Bovine Serum Albumin (BSA), Urea, and Gastric Mucin III were acquired from Sigma-Aldrich (St. Louis, MO). The 8-isoprostane ELISA kit was obtained from Cayman Chemical (Ann Arbor, MI). Potassium Chloride was purchased from JT Baker (Radnor, PA). Sodium Chloride was obtained from Research Products International (Mount Prospect, IL). Calcium Chloride, Disodium Hydrogen Phosphate, and D-(+)-Glucose were purchased from Spectrum Chemical MFG Corp (New Brunswick, NJ). Magnesium Chloride Hexahydrate was acquired from Fisher Scientific (Waltham, MA). Sodium Hydroxide was sourced from Reagents (Charlotte, NC). Hydrochloric Acid (HCl) was purchased from LabChem (Pittsburgh, PA). Alpha Amylase and Lysozyme were acquired from MP Biomedicals (Solon, OH). Acid Phosphatase was obtained from Tokyo Chemical Industry (TCI; Tokyo, Japan).

### 2.2 Preparation of AS and CAS

Preparation of AS, CAS, and CRP1.1 extract as well as extract characterization was carried out by McKinney Specialty Labs (Richmond, VA) in accordance with the International Organization for Standardization - International Electrotechnical Commission (ISO-IEC) 17025:2017 standards. The AS (Supplementary Table S1) was prepared following the procedures outlined in the German standard DIN V Test Method 53160-1 2002-10 (German Institute for Standardization, 2002). The preparation of CAS (Supplementary Table S2) was based on the method described by Chou and Hee (1994), with two modifications. Specifically, 0.68 g/L disodium hydrogen phosphate (Na_2_HPO_4_) was used instead of 0.58 g/L sodium dihydrogen phosphate (NaH_2_PO_4_); this brought the total Na^+^ concentration slightly higher to 33.5 mM. The lysozyme concentration was 17,550 U/L, which falls within the reported range for human saliva (Tomašovský et al., 2025).

### 2.3 Extraction of the CORESTA snus reference CRP1.1

**FIGURE 2:**
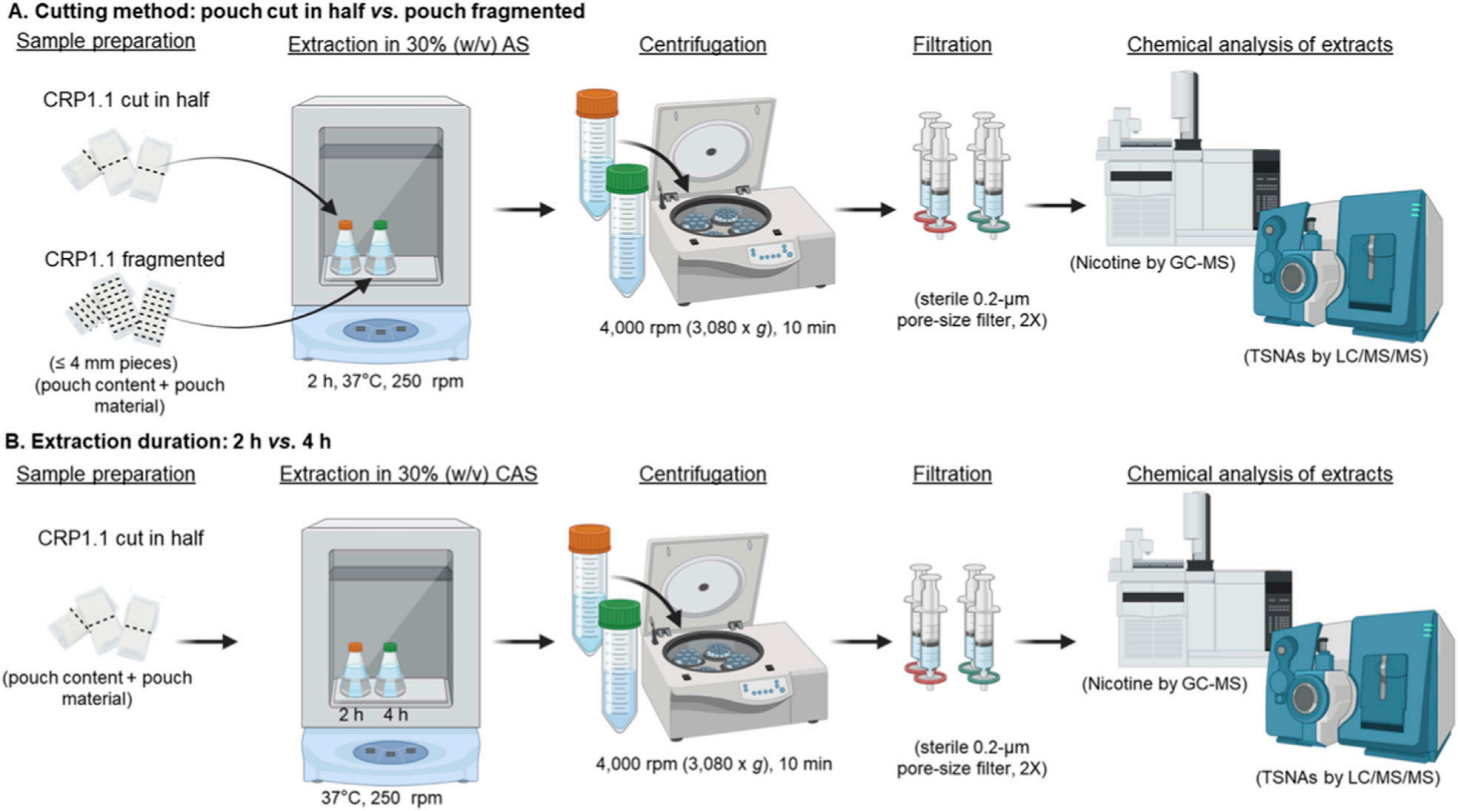
Optimization of the pouch extraction procedure. The CORESTA Swedish-style snus reference product (CRP1.1) was used as a representative oral tobacco product to optimize extraction conditions. Two parameters were evaluated: **(A)** pouch cutting method, either halving each pouch or cutting into fragments ≤4 mm pieces, and **(B)** extraction duration, comparing 2 h versus 4 h. Extraction efficiency was assessed by quantifying nicotine and TSNA concentrations.

**FIGURE 3:**
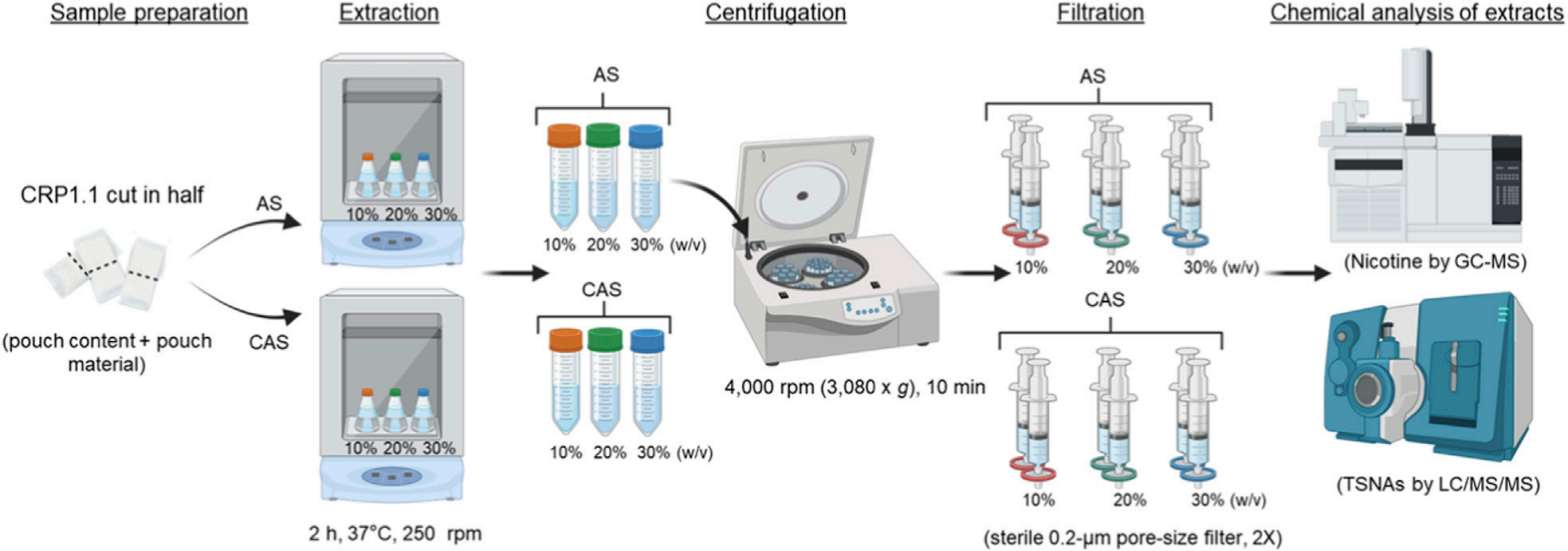
Comparative analysis of extraction efficiency across different concentrations and solvent vehicles. Using the optimized pouch extraction method, i.e., halving each pouch and a 2-h extraction time, CRP1.1 was extracted in either AS or CAS at concentrations of 10%, 20%, and 30% w/v. Extraction efficiency was assessed by quantifying nicotine and TSNA concentrations.

The CORESTA Swedish-style snus reference product, CRP1.1 (North Carolina State University, Raleigh, NC) was used as a representative oral tobacco product to optimize extraction procedures (Figure 2) and evaluate the relationship between extract concentrations and extraction efficiency (Figure 3). To refine the extraction procedures, two cutting methods were tested (Figure 2A). In the first method, each pouch was cut in half, while in the second method, each pouch was fragmented into pieces ≤4 mm. CRP1.1 (both the pouch content and wrapping material) was then extracted in freshly prepared AS at a concentration of 30% w/v. The concentrations of nicotine and TSNAs were quantified and compared across the two methods.

In addition to optimizing the pouch cutting method, the extraction duration was also refined (Figure 2B). For this purpose, CRP1.1 pouches, prepared according to the results of the cutting method optimization experiment, were suspended in 10 mL of freshly prepared CAS at a concentration of 30% w/v. The suspension was extracted for either 2 h or 4 h, followed by centrifugation and filtration. Longer extraction duration was not further tested based on previous findings where no difference in extraction efficiency was shown between 2 h and 24 h (Zhang J. et al., 2019).

Extracts of CRP1.1 were prepared in either AS or CAS at concentrations of 10%, 20%, and 30% w/v, with pouch preparation and extraction times based on the results of the optimization experiments. Briefly, the suspensions were shaken at 250 ± 25 rpm at 37 °C for 2 h, followed by centrifugation at 4,000 rpm (3,080 × *g* using an SX4400 rotor) (Beckman Coulter, Indianapolis, IN) for 10 min. The resulting supernatant was filtered once through a sterile 0.2-μm pore-size filter. For the CAS extracts at 20% and 30% w/v, two filters were required during the initial filtration. The combined filtrates were then filter-sterilized a second time using a new 0.2-μm filter unit. The final extracts were aliquoted into smaller volumes and immediately analyzed for nicotine and TSNAs.

### 2.4 Quantification of nicotine and TSNAs

Nicotine quantification was conducted using a low-nicotine method developed and validated by McKinney Specialty Labs (2022). In brief, a 100-µL aliquot of CRP1.1 extract was combined with an equal volume of 2 N sodium hydroxide in an amber screw-top bottle and allowed to stand for 15 min. Subsequently, 100 µL of nicotine-d4 (used as the internal standard) was added, followed by addition of 10 mL of methyl tert-butyl ether (MTBE). The mixture was briefly vortexed, and the layers were allowed to separate. Once separation occurred, a 100-µL aliquot of the upper organic layer was transferred to an autosampler vial and diluted with 900 µL of MTBE diluent solution. The final mixture was analyzed using gas chromatography-mass spectrometry (GC-MS; Agilent 7890 Gas Chromatograph coupled with an Agilent 5975 Mass Selective Detector; Santa Clara, CA). Nicotine separation was achieved using an Agilent J&W CAM analytical column (30 m × 0.25 mm ID; 0.25 µm df).

The quantification of TSNAs was conducted following the CORESTA CRM 72 guideline (CORESTA, 2021). A 1.0-mL aliquot of the extract was mixed with 19 mL of 100 mM ammonium acetate solution and shaken mechanically at 210 rpm to facilitate TSNA extraction. The resulting solution was filtered and analyzed using a Waters liquid chromatography system coupled with a tandem mass spectrometer (LC-MS/MS; Milford, MA). TSNA separation was achieved using a Waters Acquity BEH C18 analytical column (2.1 mm × 100 mm; 1.7 µm). Each TSNA in the sample was quantified using deuterated analogues of the analytes as internal standards, including N-Nitrosoanabasine-d4 (d4-NAB-d4), N-Nitrosoanatabine-d4 (d4-NAT), 4-(N-Nitrosomethylamino)-1-(3-pyridyl)-1-butanone-d4 (d4-NNK), and N-Nitrosonornicotine-d4 (d4-NNN).

### 2.5 *In vitro* 2D oral models and exposure

Normal human gingival fibroblasts (NHGFs) and oral epithelial cells (NHOEs) were isolated from the same donor (donor ID# G29) by MatTek Corporation and cultured as 2D monolayer cells. Cryopreserved NHGFs were recovered at passage 7, cultured in DMEM supplemented with 10% Fetal Bovine Serum (FBS) and maintained at 37 °C and 5% CO_2_ until they reached confluence. The day before the exposure, 15,000 cells were seeded into each well of a 96-well plate and incubated overnight at 37 °C and 5% CO_2_. AS and CAS were diluted to 5%, 10%, 15%, and 20% v/v in low serum media comprising DMEM and 1% FBS. Cells were exposed to the AS and CAS dilutions for 24 h at 37 °C and 5% CO_2_.

Cryopreserved NHOEs were recovered at passage 3 and cultured in EpiLife™ Medium (Gibco, Waltham, MA) and maintained at 37 °C and 5% CO_2_ until they reached confluence. The day before the exposure, 25,000 cells were seeded into each well of a 96-well plate and incubated overnight at 37 °C and 5% CO_2_. AS and CAS were diluted to 5%, 10%, 15%, and 20% v/v in EpiLife™ Medium. Cells were exposed to the AS and CAS dilutions for 24 h at 37 °C and 5% CO_2_.

### 2.6 *In vitro* 3D organotypic oral models and exposure

ORL-200 and ORL-300-FT were produced by MatTek using NHGFs and NHOEs from the same donor as those used in the 2D experiments. ORL-200 is a partial thickness oral epithelial model containing only NHOEs, while ORL-300-FT is a full thickness oral model comprising NHOEs cultured over a collagen matrix embedded with NHGFs. Both models were produced and cultured in accordance with MatTek’s internal Standard Operating Procedures. Prior to treatment, tissues were equilibrated in their respective media (i.e., ORL-200-ASY for ORL-200 and ORL-300-FT-ASY for ORL-300-FT) for approximately 1 h at 37 °C and 5% CO_2_. AS and CAS were either undiluted (100%) or diluted to 20%, 50%, and 80% v/v in TEER-Buffer-GLC (DPBS with CaCl_2_, MgCl_2_, and 3.6 mg/mL glucose; MatTek). Following the equilibration, tissues were transferred to a new 6-well plate containing 0.9 mL of fresh media. A total of 100 µL of either diluted or undiluted AS or CAS was applied to the apical surface of each tissue. Tissues were then treated for approximately 24 h.

Appropriate control groups were also included. For NHGFs and NHOEs, vehicle controls consisted of cultures maintained in the respective media. For the organotypic tissue models, controls included cultures with the apical side exposed to air and cultures exposed to TEER Buffer-GLC from the apical side. Positive controls included cultures exposed to 1% Triton X-100 (positive control for tissue death) and cultures treated with 1 μg/mL PMA/50 ng/mL ionomycin (positive control for 8-isoprostane). A 1% Triton X-100 solution is widely employed as a positive control in cell viability and cell cytotoxicity assays. It has been used by MatTek to establish the ET-50 range for EpiOral quality control (Klausner et al., 2007). The concentrations of Triton X-100 and PMA/ionomycin were selected based on the methodology reported by Keyser (2022). All control groups were treated in a similar manner and for the same duration as the AS- and CAS-exposed groups.

### 2.7 MTT viability assay

The MTT assay was conducted in accordance with MatTek’s internal SOP (Supplementary Figure S1). Following exposure, media from all samples were collected and stored at −70 °C for downstream analysis, including 8-isoprostane, IL-6, and IL-8 ELISA. The viability of NHGFs and NHOEs was subsequently assessed using the MTT assay, which measures the ability of metabolically active (viable) cells to reduce MTT dye to formazan crystals. Briefly, pre-warmed MTT working reagent (150 µL) was added to each well of the 96-well plates, and the cells were incubated with MTT reagent for 3 h at 37 °C and 5% CO_2_. At the end of the incubation, the MTT reagent was removed, and the cells were rinsed once with DPBS. Next, 150 µL of MTT extractant solution (i.e., isopropanol) was added to each well to dissolve the formazan crystals. Extraction proceeded for 2 h on a shaker at room temperature in the dark. The absorbance of the extracted samples was measured at 570 nm using a SpectraMax^®^ M2 microplate reader (Molecular Devices, San Jose, CA). Two independent experiments were performed for NHGFs and three for NHOEs, with each independent experiment comprising six technical replicates.

Following exposure, the ORL-200 and ORL-300-FT tissues were rinsed with DPBS three times, and the apical surface of the tissues was carefully dried with a sterile swab. The tissues were then transferred to a new plate containing 300 µL of pre-warmed MTT working reagent and incubated for 3 h. The tissues were blotted dry and moved to a fresh 24-well plate. MTT extractant solution (2 mL of isopropanol) was added into the apical compartment and allowed to overflow into the basolateral compartment. The tissues were extracted overnight at room temperature in the dark. At the end of the extraction period, the extractant solution from both the apical and basolateral compartments was combined and thoroughly mixed. A 200-µL aliquot of each of the extracted samples was transferred to a 96-well plate, and absorbance measured at 570 nm using a SpectraMax^®^ M2 microplate reader. Two independent experiments were performed for both ORL-200 and ORL-300-FT, with each independent experiment comprising four technical replicates.

Cell viability for treated groups was expressed as a percentage relative to the respective vehicle controls (i.e., the media- or TEER Buffer-GLC-treated groups).

### 2.8 8-Isoprostane ELISA

Media was analyzed for 8-isoprostane secretion within 10 days of collection using an 8-isoprostane ELISA kit (Cayman Chemical, Ann Arbor, MI) by following the manufacturer recommended protocol. Upon medium collection, 55 µL of the culture media from each sample was mixed with 1 µL of 2.5 mg/mL BHT to prevent oxidative formation of 8-isoprostane during storage. An eight-point standard curve of known 8-isoprostane concentrations was prepared. Then, 50 µL of each standard and culture media sample was added to a Mouse Anti-Rabbit IgG-coated ELISA plate and incubated with 50 µL of 8-isoprostane-AChE Tracer and 50 µL of 8-isoprostane ELISA Antiserum for 18 h at 4 °C. After incubation, the ELISA plate was washed and developed with Ellman’s reagent for approximately 90 min. Absorbance was measured at 405 nm using a SpectraMax^®^ M2 microplate reader. A four-parameter logistic fit was applied to the standard curve to calculate 8-isoprostane concentrations in the unknown samples. Two independent experiments were performed in NHGFs, ORL-200, and ORL-300 FT, and three for NHOEs. Each 2D cell independent experiment included six technical replicates, while each 3D tissue model experiment comprised four technical replicates.

Secretion levels of 8-isoprostane in treated groups were expressed as fold changes relative to the respective vehicle controls (i.e., the media- or TEER Buffer-GLC-treated groups), providing a normalized measure of the relative effects.

### 2.9 IL-6 and IL-8 ELISA

Following exposure, IL-6 and IL-8 concentrations in culture media were quantified using the Human IL-6 ELISA kit and Human IL-8/CXCL8 ELISA kits (R&D Systems, Minneapolis, MN) according to the manufacturer’s recommended protocol. Samples were diluted in Assay Reagent Diluent as needed prior to analysis. Briefly, 96-well microplates were coated with capture antibody overnight at room temperature. The plates were then washed and blocked with 1% BSA in PBS for 1 h at room temperature. After blocking, the plates were washed again, and 100 µL of the eight-point standard curves of known IL-6 and IL-8 concentrations, along with the unknown samples, were added to the plates and incubated for 2 h at room temperature. Following incubation, the plates were washed, and detection antibody was added for 1 h at room temperature, followed by washing and the addition of Streptavidin-HRP for 20 min. After a final wash, 100 µL of TMB Substrate Solution was added for 20 min, followed by 50 µL of 2 N H_2_SO_4_ as a stop solution. Absorbance was measured at 450 nm with a 570 nm correction using a SpectraMax^®^ M2 microplate reader. A four-parameter logistic fit was applied to the standard curve to calculate the IL-6 and IL-8 concentrations in the unknown samples. For diluted samples, the calculated concentrations were multiplied by the dilution factor prior to analysis. Two independent experiments were performed in NHGFs, ORL-200, and ORL-300 FT, and three for NHOEs. Each 2D cell independent experiment included three technical replicates, while each 3D tissue model experiment comprised four technical replicates.

Secretion levels of IL-6 and IL-8 in treated groups were expressed as fold changes relative to their respective vehicle controls (i.e., the media- or TEER Buffer-GLC-treated groups), providing a normalized measure of the relative effects.

### 2.10 Statistical analysis

The data are presented as means ± standard deviation (SD). Normality of distribution and homogeneity of variance were assessed using JMP® Pro (version 17.0.0). Normality was evaluated within the Fit Distributions platform using the Shapiro-Wilk Goodness-of-Fit Test. Homogeneity of variance was assessed with ANOM for Variances (Levene’s test based on absolute deviation from the median, ADM) within the Oneway Analysis platform. This method is robust to outliers and non-normality, enabling comparison of group variances by evaluating group ADM means against the overall ADM mean.

Treatment-related effects were analyzed using GraphPad Prism (version 10.4.3, Lo Jolla, CA). For dataset with a normal distribution, one-way ANOVA followed by Dunnett’s multiple comparison test was performed. For dataset with a non-normal distribution, the Kruskal–Wallis test followed by Dunn’s multiple comparison test was applied. Comparisons were made against the respective vehicle control, i.e., the medium-treated group for NHGFs and NHOEs, and TEER Buffer-GLC for ORL-200 and ORL-300-FT.

## 3 Results

### 3.1 Extraction efficiency comparison between AS and CAS

Before extracting CRP1.1 at various concentrations, we evaluated the effects of two key factors, i.e., cutting techniques (in AS) and extraction time (in CAS), on extraction efficiency. To examine the impact of cutting techniques, CRP1.1 pouches were either halved or fragmented into pieces of ≤4 mm before being extracted at a concentration of 30% w/v in AS. Both methods demonstrated comparable extraction efficiencies, with nicotine and TSNAs concentrations differing by less than 5%. Specifically, nicotine extraction efficiency was approximately 77%, and TSNAs extraction efficiencies ranged from 60% to 77% (NAB: ∼60%, NAT: ∼64%, NNK: ∼74%, and NNN: ∼77%). Notably, the TSNA extraction efficiency observed in this experiment was significantly higher than those in the subsequent experiments. This difference is likely due to the TSNA analytical method not being fully optimized at the time. As a result, this data is considered valid only for intra-experiment comparisons. Volume recovery was similar between the two methods, averaging around 80% (Supplementary Table S3). These results suggest that fragmenting pouches into smaller pieces does not improve extraction efficiency further. Halving the pouch, therefore, was selected for subsequent studies.

To investigate the impact of extraction time, CRP1.1 cut in half was suspended in CAS at a concentration of 30% w/v and extracted for either 2 h or 4 h. Both extraction durations produced similar extraction efficiencies, with nicotine and TSNAs concentrations differing by less than 8%. Nicotine extraction efficiency was approximately 70%, and TSNAs extraction efficiency ranged from 47% to 67% (NAB: ∼50%, NAT: ∼47%, NNK: ∼50%, and NNN: ∼67%) between the two time points. Volume recovery was also comparable between the two durations, averaging around 40% (Supplementary Table S4). However, the volume recovery rate was lower than that observed in the other two experiments. This reduction may be attributed to two factors. At the end of the extraction, a significant amount of extract was retained by the filter disc. Additionally, the extraction was conducted in a relatively smaller volume (10 mL). Despite the lower recovery rate, the data are considered valid for intra-experiment comparison. These findings indicate that extending the extraction duration does not improve extraction efficiency further. Therefore, the 2-h extraction duration was selected for subsequent studies.

The difference in extraction efficiency between AS and CAS was examined by extracting CRP1.1 in both solvent vehicles at concentrations of 10%, 20%, and 30% w/v using optimized procedures as described above. Overall, a decrease in nicotine and TSNA extraction efficiency was observed in both AS and CAS extracts as extract concentrations increased (Table 1). Nicotine extraction efficiency decreased from approximately 83% at 10% w/v to 66% at 30% w/v in both AS and CAS. However, the reduction in TSNA extraction efficiencies was more pronounced in AS (dropping from approximately 85%–90% to around 50%) compared to in CAS (declining from approximately 60%–67% to 45%–60%). Notably, TSNA extraction efficiencies in AS extracts were consistently higher at 10% and 20% w/v concentrations compared to those in CAS extracts. However, both solvent vehicles performed similarly at 30% w/v. In addition, volume recovery was comparable between AS and CAS extracts, with both solvent vehicles exhibiting a decreasing trend from 88% at 10% w/v to 74% at 30% w/v.

**TABLE 1:** Concentrations and extraction efficiency of nicotine and select TSNAs in CRP1.1 extract in AS and CAS.

Extract Conc. (w/v)		Nicotine		TSNAs								Volume recovery (%)
				NAB		NAT		NNK		NNN		
		Conc. (mg/g)	Extraction efficiency (%)^a^	Conc. (ng/g)	Extraction efficiency (%)^a^	Conc. (ng/g)	Extraction efficiency (%)^a^	Conc. (ng/g)	Extraction efficiency (%)^a^	Conc. (ng/g)	Extraction efficiency (%)^a^	
AS	10%	6.50 (0.05)	85.53	7.68 (0.34)	85.33	121 (2)	86.41	48 (0.26)	92.31	172 (5)	90.53	82.1
	20%	5.97 (0.03)	78.55	5.64 (0.44)	62.67	92.0 (9.0)	65.71	39.0 (4.5)	75.00	146 13)	76.84	85.3
	30%	5.01 (0.03)	65.92	4.22 (0.29)	46.89	65.7 (2.9)	46.93	27.9 (1.5)	53.65	108 (6)	56.84	75.5
CAS	10%	6.31 (0.03)	83.03	<LOQ	NA	83.4 (3.0)	59.57	32.5 (1.7)	62.5	127 (10)	66.84	88.0
	20%	5.60 (0.06)	73.68	4.86 (0.90)	54.00	72.2 (10.6)	51.57	32.3 (6.1)	62.12	122 (18)	64.21	74.8
	30%	5.03 (0.06)	66.18	4.31 (0.1)	47.9	61.6 (3.8)	44.00	28.0 (3.0)	53.85	113 (10)	59.47	73.8

^a^ % calculated from mean value reported in the CORESTA, 2017 study.

% Extraction efficiency is calculated relative to CORESTA, reference data and based on the assumption that CORESTA, reference data represents 100% recovery of the respective analytes.

LOQ, level of quantification; NA, not applicable.

Data is expressed as mean (SD).

### 3.2 Effects of AS and CAS on cell viability

**FIGURE 4:**
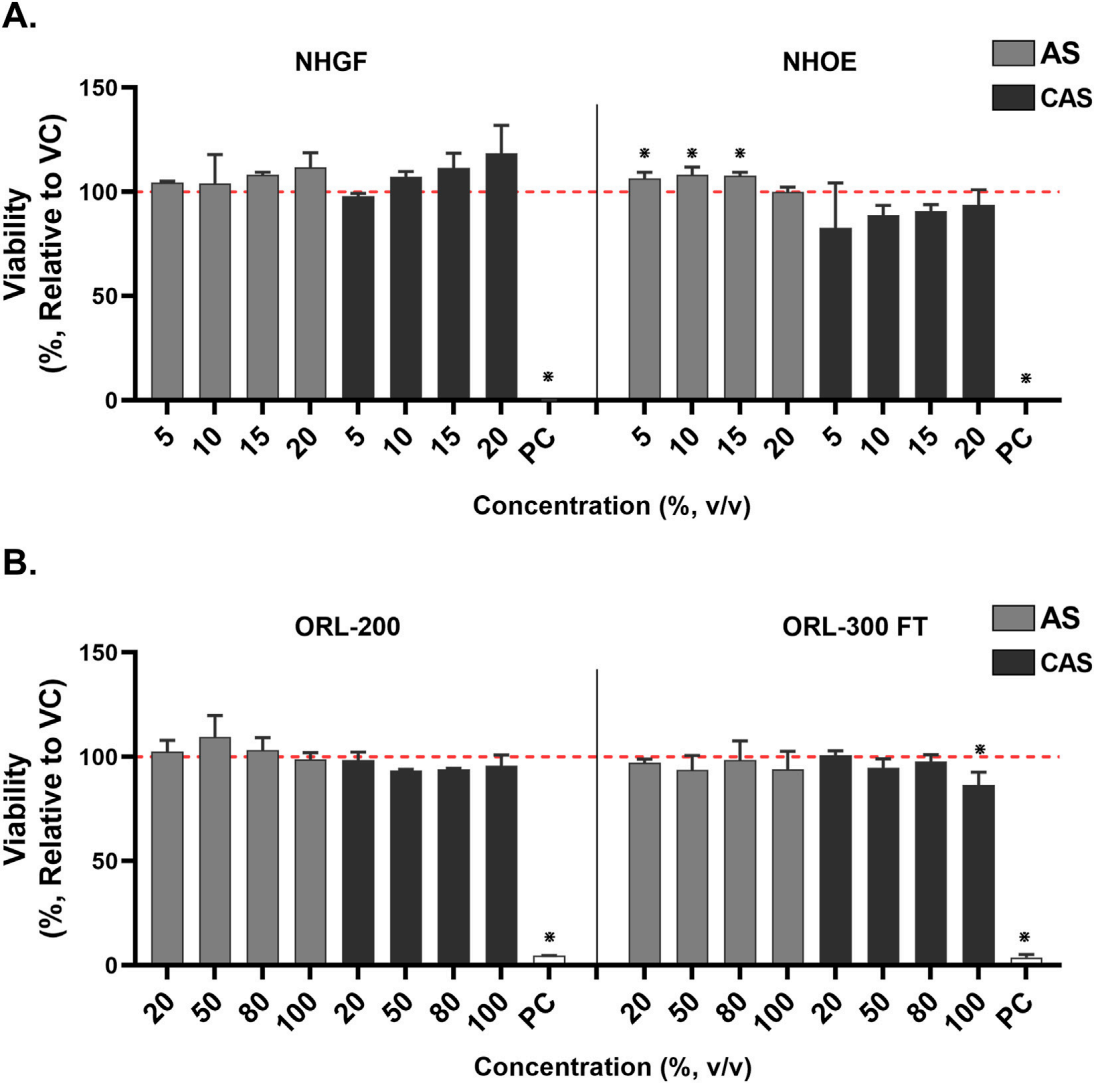
Cytotoxicity of AS and CAS across four *in vitro* models. Cytotoxicity was assessed using the MTT viability assay in 2D cells **(A)** and 3D models **(B)**. Data (*n* = 2 for NHGFs and the 3D models; *n* = 3 for NHOEs) are expressed as Means ± SD. *n* is the number of independent experiments. Each independent experiment contained 6 technical replicates in the 2D experiments and 4 technical replicates in the 3D experiments. 1% Triton X-100 was included as the positive control (PC). Vehicle control values are shown as a red dotted line. *p < 0.05 is considered statistically significant compared to the corresponding experimental vehicle controls.

Cell viability was assessed using the MTT assay 24 h after treatment (Figure 4). The viability of AS-treated NHGFs and NHOEs consistently remained around 100% (Figure 4A, grey bars). While a statistically significant increase in viability was observed from 5% to 15% v/v of AS in NHOEs, the change was marginal (less than 10%) and therefore not considered biologically relevant. Similarly, the viability changes in CAS-treated NHGFs and NHOEs were also within 10%, except at 5% v/v of CAS in NHOEs (Figure 4A, black bars). However, none of these responses reached statistical significance.

After 24 h of exposure, the viability of AS-exposed ORL-200 and ORL-300-FT tissue models remained above 90% at all test concentrations (Figure 4B, grey bars). Similarly, the viability of both organotypic tissue models exposed to CAS was above 90%, except for the ORL-300-FT model at 100% CAS (Figure 4B, black bars). The reduction in viability observed at 100% CAS in this model was statistically significant. However, this decrease was less than 15%, suggesting that it may not have a meaningful biological impact.

### 3.3 Effects of AS and CAS on 8-isoprostane release

**FIGURE 5:**
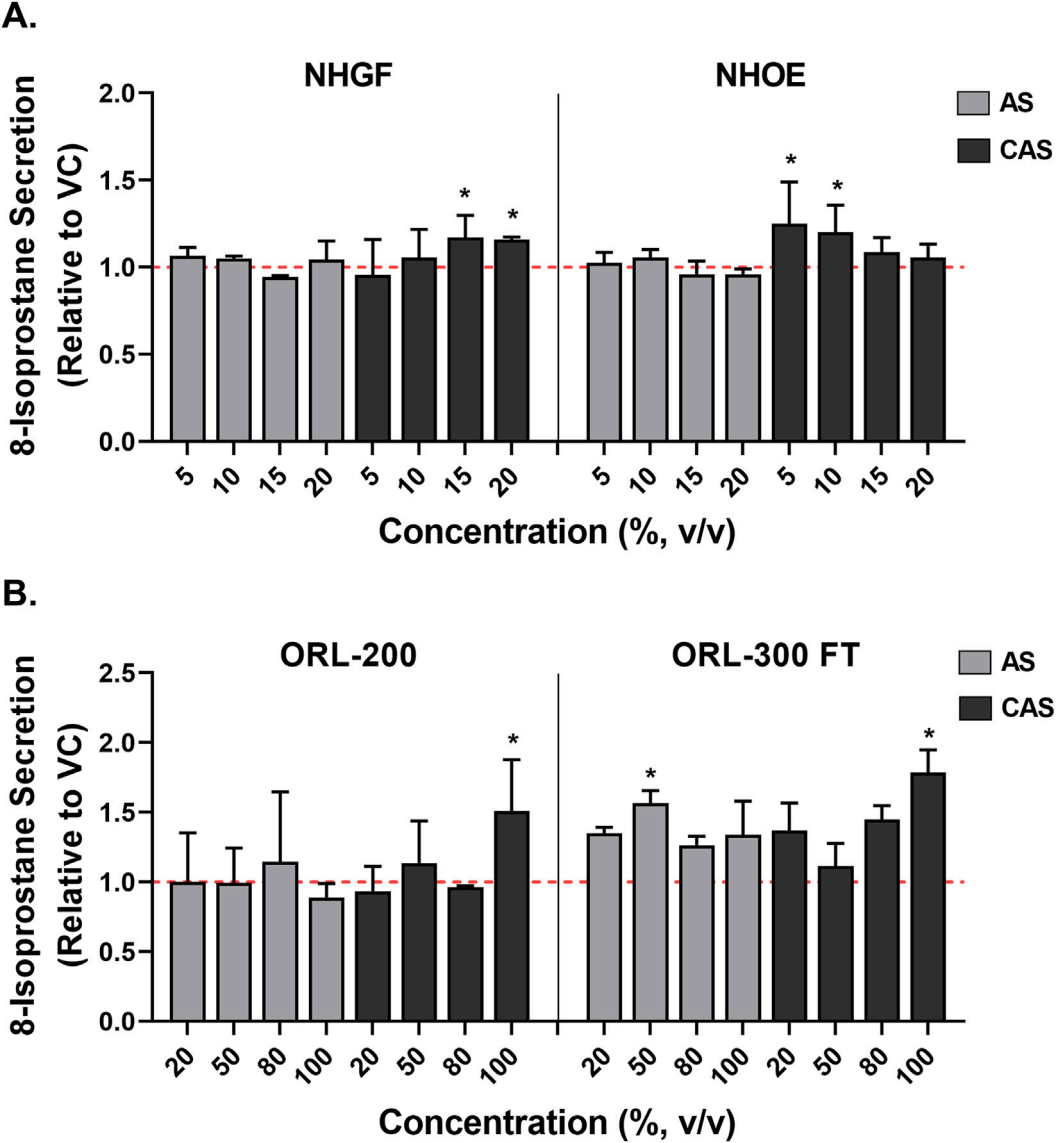
Oxidative stress induced by AS and CAS across four *in vitro* models. Induction of oxidative stress was assessed by measuring the secretion of 8-isoprostane in 2D cells **(A)** and 3D models **(B)**. Data (*n* = 2 for NHGFs and the 3D models; *n* = 3 for NHOEs) are expressed as Means ± SD. *n* is the number of independent experiments. Each independent experiment contained 6 technical replicates in the 2D experiments and 4 technical replicates in the 3D experiments. PMA/ionomycin was used as the positive control for 8-isoprostane secretion. In NHGFs and NHOEs, it induced approximately 65-fold and 2-fold increases, respectively. In ORL-200 and ORL-300 FT, the increases were approximately 10-fold and 35-fold, respectively. Vehicle control values are shown as a red dotted line. *p < 0.05 is considered statistically significant compared to the corresponding experimental vehicle controls.

Oxidative stress induction was assessed by measuring 8-isoprostane secretion in the culture media 24 h post-exposure. To facilitate comparisons across different *in vitro* models, 8-isoprostane levels were normalized to their respective vehicle controls. In NHGFs and NHOEs, exposure to AS did not alter 8-isoprostane levels (Figure 5A, grey bars). In contrast, CAS exposure increased 8-isoprostane secretion by approximately 15% at the two highest concentrations in NHGFs (Figure 5A, left panel, black bars). In NHOEs, CAS exposure resulted in an approximately 20% increase in 8-isoprostane release at the two lowest concentrations (Figure 5A, right panel, black bars). These CAS-induced responses in both cell type were statistically significant.

In ORL-200, neither AS nor CAS exhibited a dose-dependent effect on 8-isoprostane release, although CAS induced a statistically significant increase of approximately 50% at a concentration of 100% (Figure 5B, left panel). However, in the ORL-300-FT model, AS exposure led to an approximately 50% increase in 8-isoprostane secretion at a concentration of 50% v/v (Figure 5B, right panel, grey bars). Given the absence of a dose-response relationship, this increase is likely attributable to intrinsic variability within the organotypic tissue models. In contrast, CAS exposure induced a significant increase of approximately 1.8-fold in 8-isoprostane secretion at a concentration of 100% v/v in the ORL-300-FT model (Figure 5B, right panel, black bars).

### 3.4 Effects of AS and CAS on the secretion of IL-6 and IL-8

**FIGURE 6:**
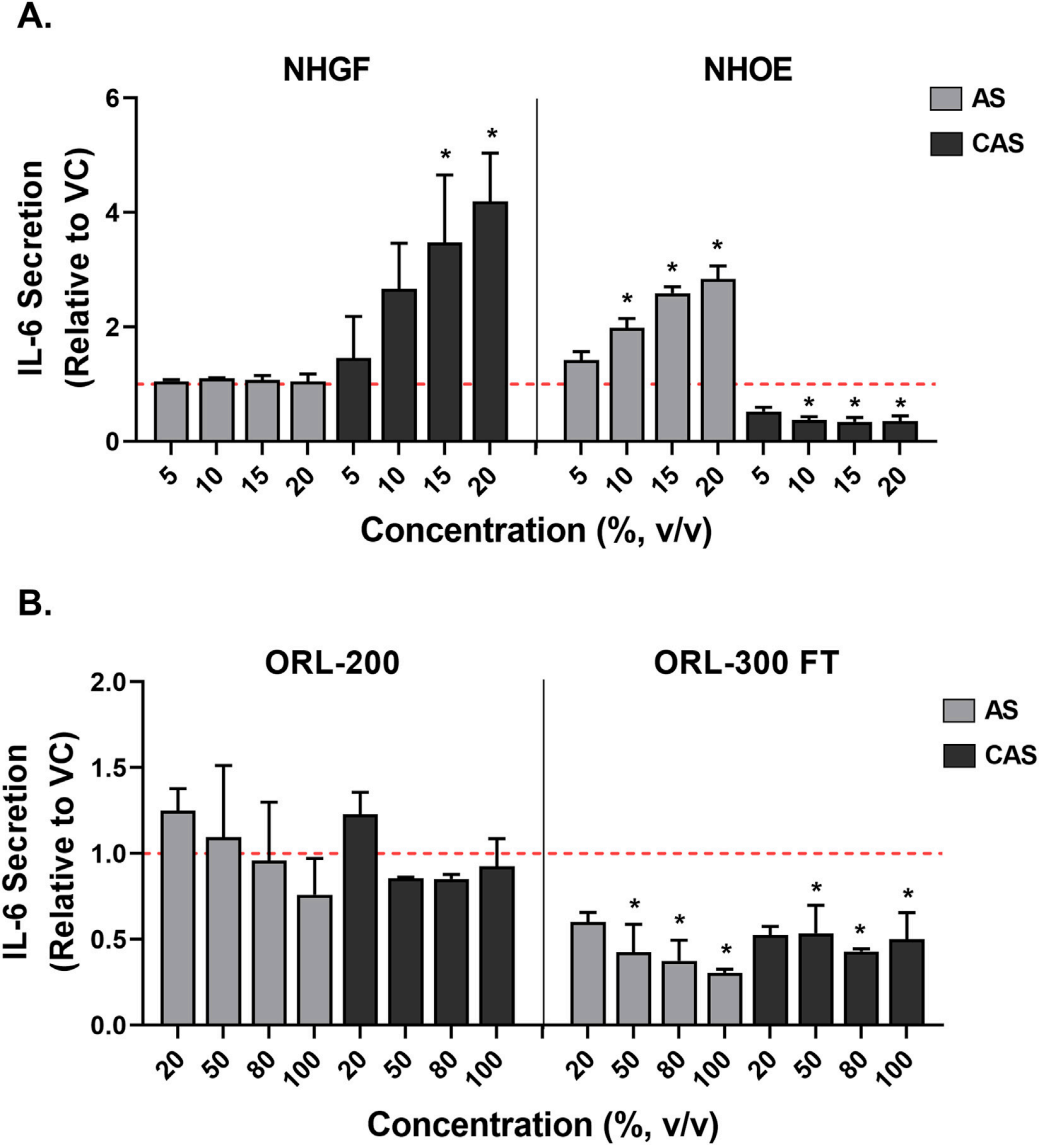
Modulation of IL-6 secretion by AS and CAS across four *in vitro* models. Modulation of IL-6 secretion was evaluated in 2D cells **(A)** and 3D models **(B)**. Data (*n* = 2 for NHGFs and the 3D models; *n* = 3 for NHOEs) are expressed as Means ± SD. *n* is the number of independent experiments. Each independent experiment contained 3 technical replicates in the 2D experiments and 4 technical replicates in the 3D experiments. Vehicle control values are shown as a red dotted line. *p < 0.05 is considered statistically significant compared to the corresponding experimental vehicle controls.

The modulation of inflammatory responses by AS and CAS was evaluated by measuring the release of IL-6 and IL-8 into the media during a 24-h exposure. The effects of AS and CAS on IL-6 secretion differed between the two cell types. In NHOEs, AS and CAS had opposing effects: AS significantly increased IL-6 secretion, while CAS inhibited it (Figure 6A, right panel). In NHGFs, CAS elevated IL-6 release in a concentration-dependent manner (Figure 6A, left panel, black bars). In contrast, in ORL-300 FT, both AS and CAS significantly suppressed IL-6 secretion, suggesting potential anti-inflammatory effects (Figure 6B, right panel). Neither AS nor CAS induced significant changes in ORL-200 (Figure 6B, left panel).

**FIGURE 7:**
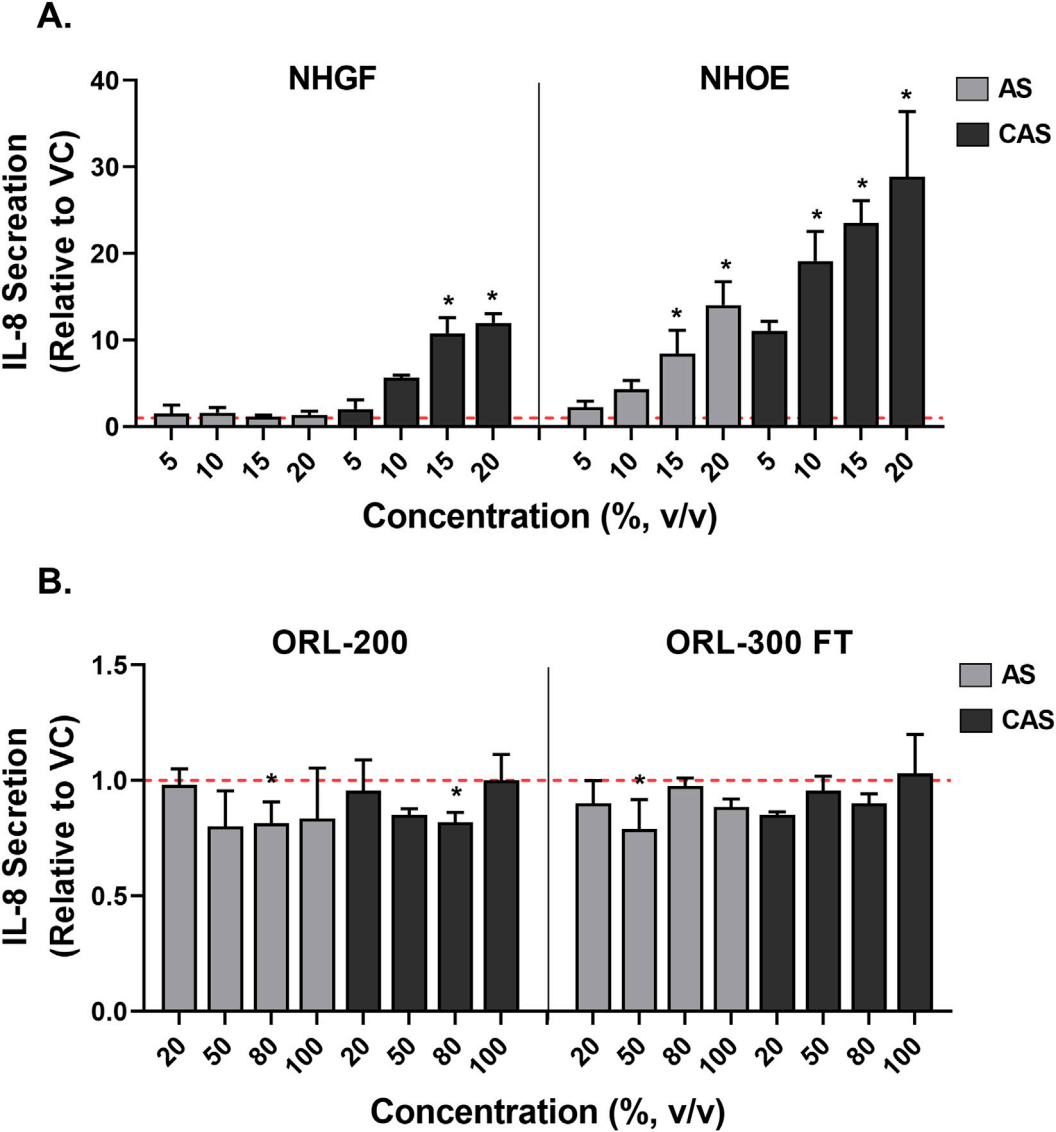
Modulation of IL-8 secretion by AS and CAS across four *in vitro* models. Modulation of IL-8 secretion was evaluated in 2D cells **(A)** and 3D models **(B)**. Data (*n* = 2 for NHGFs and the 3D models; *n* = 3 for NHOEs) are expressed as Means ± SD. *n* is the number of independent experiments. Each independent experiment contained 3 technical replicates in the 2D experiments and 4 technical replicates in the 3D experiments. Vehicle control values are shown as a red dotted line. *p < 0.05 is considered statistically significant compared to the corresponding experimental vehicle controls.

The impacts of AS and CAS exposure on IL-8 secretion also varied across the tested models. AS markedly increased IL-8 release in NHOEs by up to 15-fold in a concentration-dependent manner (Figure 7A, right panel, grey bars). CAS significantly stimulated IL-8 secretion in both NHGFs and NHOEs, with a more pronounced effect observed in NHOEs compared to NHGFs (Figure 7A, left panel vs. right panel, black bars). However, neither AS nor CAS significantly modulated IL-8 secretion in the organotypic tissue models under the tested conditions (Figure 7B). Even though some responses reached statistical significance, the changes are likely due to intrinsic variability within the organotypic tissue models, given the absence of a dose-response relationship.

## 4 Discussions

In this study, we assessed the key toxicological characteristics of AS and CAS using four *in vitro* oral models, ranging from 2D monolayer primary cells to 3D organotypic tissue models. In addition to the biological assessment, we conducted an analytical evaluation using the CORESTA ST reference product CRP1.1 as a representative oral tobacco product and quantified the levels of nicotine and TSNAs extracted into AS and CAS at varying extract concentrations. By integrating biological and analytical characterization, this study establishes a robust and comprehensive framework for identifying the suitable test system for future mechanistic investigations of oral tobacco products, including OTDNs.

The chemical compositions of AS (German Institute for Standardization, 2002) and CAS (Chou and Hee, 1994; Keyser, 2022) are shown in Supplementary Tables S1 and S2, respectively. Both are buffered solutions formulated to simulate human saliva. While their inorganic components largely overlap, they differ in concentration. In addition to inorganic salts, CAS contains key salivary constituents, such as enzymes, mucins, glucose, and urea, at concentrations within the range reported for human saliva (Keyser, 2022), making it a closer approximation of natural saliva compared to AS.

In the chemical characterization study, extraction efficiency for both nicotine and TSNAs decreased as extract concentrations increased. These trends suggest potential saturation effects or reduced solubility at higher extract concentrations. Additionally, changes in viscosity at higher extract concentration may hinder mass transfer, further lowering extraction efficiency. When comparing TSNA extraction efficiencies between AS and CAS, AS extracts consistently showed higher values at 10% and 20% w/v than CAS extracts. This difference may be attributed to protein-based components in CAS, such as mucins or enzymes, which could bind or adsorb TSNAs, thereby reducing their free concentrations available for quantification. For instance, mucins have been shown to form non-covalent interactions with small molecules and proteins through electrostatic forces, hydrogen bonding, and hydrophobic interactions (Çelebioğlu et al., 2020; Senapati et al., 2010). Such interactions are well-documented in studies demonstrating that polyphenols from tea and wine bind to salivary proline-rich proteins and mucins, a mechanism underlying the sensation of astringency (Brown et al., 2021). Our findings demonstrate that the biological effects of AS and CAS are most evident in endpoints related to oxidative stress and inflammatory responses, parameters often overlooked in conventional vehicle control studies. Notably, significant differences in inflammatory responses were observed between 2D and 3D organotypic oral models, underscoring the need to evaluate endpoints beyond cytotoxicity when assessing the baseline biological effects of solvent vehicles in *in vitro* mechanistic testing. Based on our observations, CAS and the ORL-300-FT oral mucosal model were selected as the preferred test system for future OTDN product testing, owing to their minimal solvent vehicle effects and greater biological relevance.

The use of ST has been linked to the onset and progression of various oral diseases, including periodontal disease and oral cancer (Quadri et al., 2024; Siddiqi et al., 2020). Periodontal disease is a chronic inflammatory condition characterized by the gradual destruction and loss of periodontal tissues. Its development and progression are closely associated with oxidative stress and inflammatory responses, which may be either triggered by oxidative stress itself or exacerbated by oral pathogens (Kajiya and Kurihara, 2021; Patil et al., 2024). Unlike ST products, OTDN products do not contain tobacco leaf and typically have fewer or significantly lower levels of HPHCs. However, the impact of OTDN products on oral health remains poorly understood (Jackson et al., 2023). Some localized oral mucosal changes, such as leukoplakia lesions, intraepithelial and connective tissue oedema, and immune cell infiltration, have been reported and associated with OTDN use (Miluna-Meldere et al., 2024). Despite the reported adverse changes in the oral cavity, a recent study by Liu et al. (2025) demonstrated improvements in gingival health among smokers who transitioned to OTDN products. Given the limited information regarding the oral health effects of OTDN products, this study focused on key endpoints related to ST-associated oral diseases and evaluated the effects of AS and CAS on the baseline of these endpoints.

The selection of an appropriate solvent vehicle is essential for ensuring the accuracy and biological relevance of experimental findings in toxicological studies. AS, an enzyme-free solution, is widely used in studies evaluating the effects of oral exposure to chemicals, pharmaceuticals, and tobacco products due to its simplicity and ease of use (Ali et al., 2021; Li et al., 2013; Pytko-Polonczyk et al., 2017). However, the absence of key biological components, such as mucin, proteins, and salivary enzymes, may limit its ability to faithfully replicate the physiological environment of the oral cavity, potentially influencing the outcomes of *in vitro* experiments. To address this limitation, CAS, which more closely mimics the composition and concentrations of human saliva, is considered a more physiologically relevant saliva substitute (Chou and Hee, 1994; Keyser, 2022; Malpass et al., 2013).

Our data indicate that the compositions of the solvent vehicles, particularly the presence of proteins, can significantly influence cellular responses. While neither AS nor CAS exhibited cytotoxicity in any of the four *in vitro* oral models, both elicited inflammatory effects in NHGFs and NHOEs, as evidenced by increased release of IL-6 and IL-8. The pro-inflammatory responses observed with CAS in our study are consistent with findings in normal adult human dermal fibroblasts, where significant changes in IL-6, IL-8, and vascular cell adhesion molecule 1, were detected at either the gene or protein levels (Malpass et al., 2013). Malpass et al. further demonstrated that enzymatically active ɑ-amylase, the most abundant protein in human saliva, is the primary driver of these pro-inflammatory responses. ɑ-Amylase catalyzes the breakdown of starch and glycogen into maltose, leading to elevated glucose levels in the cytosol (Papacosta and Nassis, 2011). This ɑ-amylase-catalyzed hyper-glucose condition has been shown to trigger pro-inflammatory responses in human macrophages (Price et al., 2020). However, this hypothesis has not yet been tested in NHGFs or NHOEs. Additionally, due to its role in promoting inflammation, ɑ-amylase has been investigated as a potential biomarker for periodontitis in humans (Hashim et al., 2024). These findings highlight the importance of assessing the impact of solvent vehicles in mechanistic toxicology research.

In contrast to their effects on 2D monolayer cells, both AS and CAS exhibited either anti-inflammatory responses, as indicated by reduced IL-6 secretion, or no inflammatory responses, as evidenced by unchanged IL-8 secretion, in 3D oral mucosal models. These differential responses between 2D monolayer cells and 3D mucosal tissue models may be attributed to differences in receptor expression involved in inflammatory signaling or in tissue barrier properties that are enhanced by cell-cell interactions in the 3D oral mucosal models (Yadev et al., 2011; Klausner et al., 2021). For instance, Pinnock et al. (2014) reported notable differences in inflammatory responses to *P. gingivalis* infection between 2D and 3D oral mucosal models, with pro-inflammatory responses being more pronounced in the 3D models. Given that ɑ-amylase is the most abundant protein in human saliva and the healthy oral cavity is typically in a non-inflamed state, the inflammatory effects of AS and CAS observed in 3D oral mucosal models are likely to more accurately reflect human physiological responses.

Our study highlights the importance of thoroughly evaluating the effects of solvent vehicles in *in vitro* testing. Assessing responses beyond cytotoxicity is crucial, particularly in mechanistic investigations that focus on non-traditional, disease-relevant endpoints. However, certain limitations of our study should be acknowledged. First of all, we focused exclusively on cytotoxicity, oxidative stress, and inflammatory responses. While these endpoints provide valuable insights, the broad potential for mechanistic testing suggests that future studies should expand the scope of toxicity assessment for solvent vehicles by incorporating a wider range of endpoints. Such an approach would enable a more comprehensive understanding of the baseline effects of AS and CAS across different *in vitro* models.

Another limitation pertains to the experimental vehicle control used in this study. Culture medium and TEER Buffer-GLC were employed as experimental vehicle controls for diluting AS and CAS in 2D monolayer cells and 3D oral mucosal models, respectively. While this approach is less concerning in 2D cells, it presents challenges in 3D mucosal models. Specifically, the treatment solutions for the experimental vehicle control group and the 100% AS or CAS treatment groups were greatly different, even though the effects of AS and CAS were evaluated relative to the experimental control. Additionally, covering the apical surface of the 3D oral mucosal models significantly altered biological responses compared to the air-exposed group. This observation is not unprecedented. It has been reported that applying liquid to the apical surface of organotypic airway models, which are similar to air-liquid-interface tissue models, can lead to changes in biological pathway activity and epithelial barrier integrity (Mallek et al., 2024). These discrepancies represent inherent limitations that cannot be fully addressed. Nevertheless, we believe these limitations do not compromise the validity of the study’s conclusions regarding the 3D oral mucosal models.

In conclusion, our findings support the use of CAS and the ORL-300-FT model for future mechanistic investigation of oral tobacco products, given their minimal solvent vehicle effects and enhanced biological relevance. Notably, 100% CAS induced an approximately 1.8-fold increase in 8-isoprostane secretion in ORL-300-FT models. While this increase is statistically significant, it remains within a tolerable threshold for biological response. The selection of the ORL-300-FT model was based on its overall performance across all endpoints, as well as its greater biological relevance in tissue morphology and functional responses compared to 2D cell models and the ORL-200 model. The ORL-300-FT models effectively mimic both the oral epithelium and the underlying stroma. The inclusion of fibroblasts is crucial as they are known to play a vital role in supplying cellular factors necessary for keratinocytes to mature into squamous epithelium (Russo et al., 2020; Maruguchi et al., 1994). Additionally, Yadev et al. (2011) demonstrated that full-thickness oral models, compared to partial-thickness models, are not only histologically more similar to normal human oral mucosa but also exhibit immune responses that more closely resemble those of native tissues following infection with *C. albicans*. These findings highlight the advantage of incorporating stromal cells in *in vitro* mechanistic studies. The integration of CAS with the ORL-300-FT model is anticipated to significantly enhance *in vitro* mechanistic testing of tobacco products, offering a more robust and biologically relevant platform to advance future research in this field.

## Data Availability

The original contributions presented in the study are included in the article/Supplementary Material, further inquiries can be directed to the corresponding author.
