# Supplementary material for: Comparative evaluation of artificial saliva and complete artificial saliva as solvent vehicles for in vitro toxicity testing of oral tobacco products
Source: Front Toxicol. 2025 Dec 8;7:1657073. doi: 10.3389/ftox.2025.1657073 (PMC12719288; doi:10.3389/ftox.2025.1657073)
Supplement: Supplementary file 1 [file DataSheet1.docx]

**Supplementary Materials**

**Comparative Evaluation of Artificial Saliva and Complete Artificial Saliva as Solvent Vehicles for In Vitro Toxicity Testing of Oral Tobacco Products**

**Xuefei Cao^1*^_,_ Mariana T. Farcas^1^, Yevgeniya V. Prepelitskaya^1^, Jennifer Molignano^2^, Jonathan Oldach^2^, Marisol M. Gutierrez^1^**

**^1^Altria Client Services LLC, 601 E. Jackson St., Richmond, VA 23219, USA**

**^2^MatTek Corporation, 200 Homer Ave, Ashland, MA 01721**

*To whom correspondence should be addressed at:

Xuefei Cao

Altria Client Services LLC

601 E. Jackson St., Richmond, VA 23219, USA

email: Xuefei.cao@altria.com

**Table S1. Composition of artificial saliva (AS).**

| **Source** | **Chemical Abstracts Service**  **Registry Number**  **(CAS RN #)** | **Molecular Weight (MW)** | **Concentration (g/L)** |
| --- | --- | --- | --- |
| Potassium Phosphate Dibasic, Anhydrous | 7758-79-4 | 141.96 | 0.676 |
| Potassium Chloride | 7447-40-7 | 74.55 | 0.750 |
| Sodium Chloride | 7647-14-5 | 58.44 | 0.326 |
| Calcium Chloride, Anhydrous | 10043-52-4 | 110.98 | 0.150 |
| Magnesium Chloride Hexahydrate | 7791-18-6 | 203.30 | 0.176 |
| Potassium Carbonate | 584-08-7 | 138.21 | 0.528 |

**Table S2. Composition of complete artificial saliva (CAS).**

| **Source** | **Chemical Abstracts Service**  **Registry Number**  **(CAS RN #)** | **Molecular Weight (MW)** | **Concentration (g/L)** |
| --- | --- | --- | --- |
| Potassium Chloride | 7447-40-7 | 74.55 | 0.950 |
| Sodium Chloride | 7647-14-5 | 58.44 | 1.400 |
| Calcium Chloride, Anhydrous | 10043-52-4 | 110.98 | 0.270 |
| Potassium Phosphate Dibasic, Anhydrous | 7758-79-4 | 141.96 | 0.680 |
| Magnesium Chloride Hexahydrate | 7791-18-6 | 203.30 | 0.210 |
| Urea | 57-13-6 | N/A^†^ | 0.090 |
| D-(+)-Glucose | 50-99-7 | N/A^†^ | 0.200 |
| Gastric Mucin III | N/A* | N/A^†^ | 2.700 |
| Alpha-amylase | N/A* | N/A^†^ | 100,000 U/L |
| Lysozyme | N/A* | N/A^†^ | 17,550 U/L |
| Acid Phosphatase | N/A* | N/A^†^ | 4 U/L |

Note: Not applicable (N/A*): Heterogenous mixture, CAS registry number is not applicable. N/A^†^:  Molecular Weight (MW) depending on the source (see Reagents section).

**Table S3. Concentrations and extraction efficiencies of nicotine and select TSNAs in 30% AS CRP1.1 extract under two pouch cutting methods. Data is expressed as mean (SD).**

| **Cutting Method** | **Nicotine** | | **TSNAs** | | | | | | | | **Volume Recovery (%)** |
| --- | --- | --- | --- | --- | --- | --- | --- | --- | --- | --- | --- |
|  |  |  | **NAB** | | **NAT** | | **NNK** | | **NNN** | |  |
|  | Conc. (mg/g) | Extraction Efficiency (%)^a^ | Conc. (ng/g) | Extraction Efficiency (%)^a^ | Conc. (ng/g) | Extraction Efficiency (%)^a^ | Conc. (ng/g) | Extraction Efficiency (%)^a^ | Conc. (ng/g) | Extraction Efficiency (%)^a^ |  |
| **Cut in half** | 1.72 (0.0076) | 76.7 | 5.27 (0.23) | 58.6 | 86.9 (4.54) | 62.1 | 37.50 (1.34) | 72.1 | 147.00  (3.43) | 77.4 | 83 |
| **Fragmentation** | 1.73 (0.0138) | 77.0 | 5.49 (0.31) | 61.0 | 90.70 (7.27) | 64.8 | 38.60 (1.48) | 74.2 | 146 (5.49) | 76.8 | 79 |

a: % calculated from mean value reported in the CORESTA 2017 study.

% Extraction efficiency is calculated relative to CORESTA reference data and based on the assumption that CORESTA reference data is 100% accurate.

**Table S4. Concentrations and extraction efficiencies of nicotine and TSNAs in 30% CAS CRP1.1 extract at two extraction durations. Data is expressed as mean (SD).**

| **Extraction Duration** | **Nicotine** | | **TSNAs** | | | | | | | | **Volume Recovery (%)** |
| --- | --- | --- | --- | --- | --- | --- | --- | --- | --- | --- | --- |
|  |  |  | **NAB** | | **NAT** | | **NNK** | | **NNN** | |  |
|  | Conc. (mg/g) | Extraction Efficiency (%)^a^ | Conc. (ng/g) | Extraction Efficiency (%)^a^ | Conc. (ng/g) | Extraction Efficiency (%)^a^ | Conc. (ng/g) | Extraction Efficiency (%)^a^ | Conc. (ng/g) | Extraction Efficiency (%)^a^ |  |
| **2 h** | 5.20 (0.113) | 68.5 | 4.46 (0.191) | 49.6 | 66.39 (4.21) | 47.4 | 26.30 (1.93) | 50.6 | 125.94 (4.35) | 66.3 | 40.0 |
| **4 h** | 5.33 (0.0876) | 70.1 | 4.65 (0.118) | 51.7 | 66.46 (2.38) | 47.5 | 25.80 (0.947) | 50.6 | 128.28 (5.73) | 67.5 | 38.8 |

a: % calculated from mean value reported in the CORESTA 2017 study.

% Extraction efficiency is calculated relative to CORESTA reference data and based on the assumption that CORESTA reference data is 100% accurate.

**
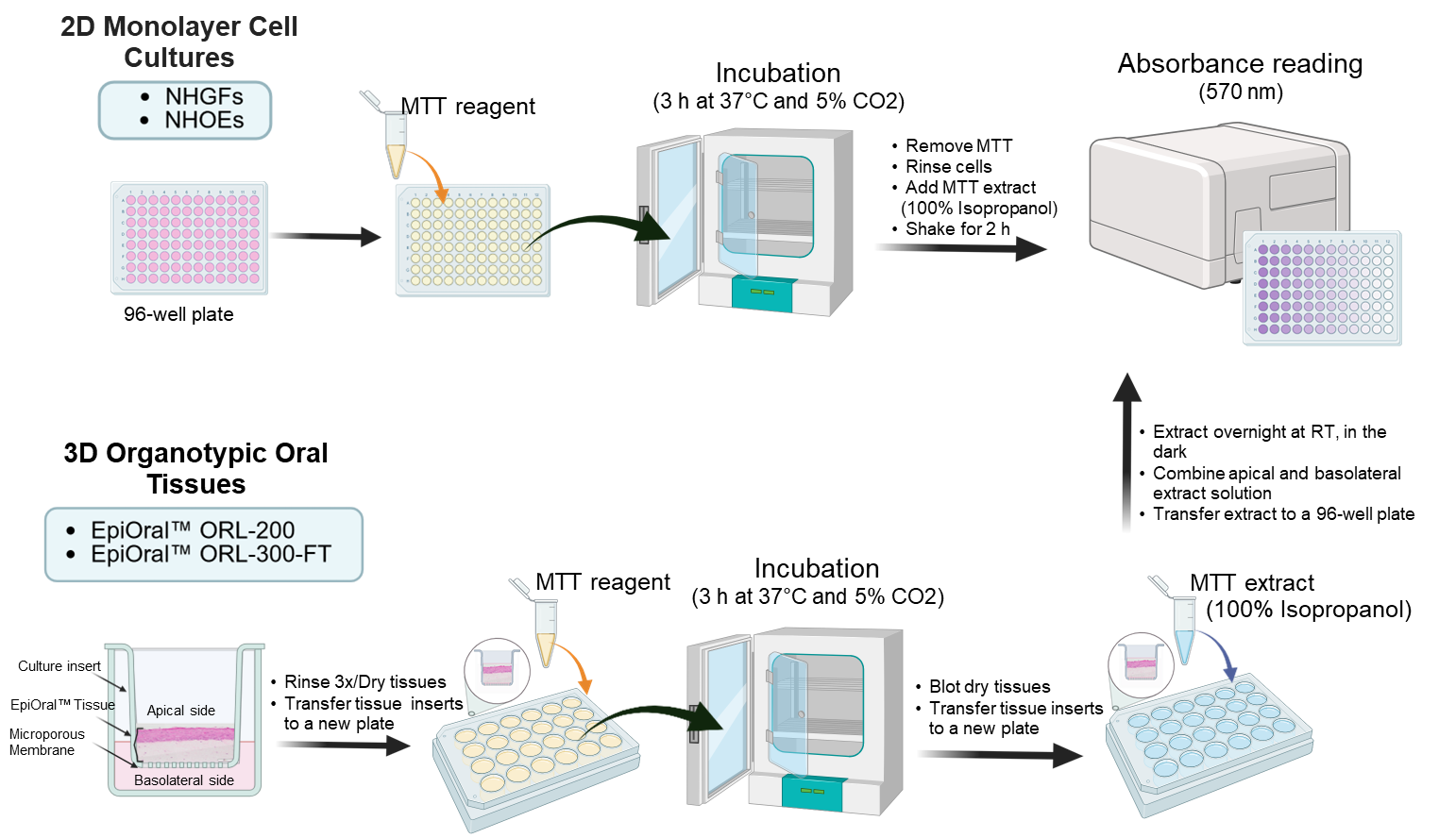
**

**Figure S1. Diagram illustrating the MTT assay workflow for assessing cell viability.** Two experimental set-ups were employed: 2D monolayer cultures and 3D organotypic oral tissues. The assay was performed in accordance with MatTek’s internal SOP.
